# Unencapsulated thymoma: a case report

**DOI:** 10.1093/jscr/rjad543

**Published:** 2023-10-14

**Authors:** Saki Yamamoto, Eiki Mizutani, Riichiro Morita, Makoto Kodama, Keiko Abe, Takuya Yazawa

**Affiliations:** Department of Thoracic Surgery, Tokyo Yamate Medical Center, 3-22-1 Hyakunin-cho, Shinjuku-ku, Tokyo, 169-0073, Japan; Department of Thoracic Surgery, Tokyo Yamate Medical Center, 3-22-1 Hyakunin-cho, Shinjuku-ku, Tokyo, 169-0073, Japan; Department of Thoracic Surgery, Tokyo Yamate Medical Center, 3-22-1 Hyakunin-cho, Shinjuku-ku, Tokyo, 169-0073, Japan; Department of Pathology, Tokyo Yamate Medical Center, 3-22-1 Hyakunin-cho, Shinjuku-ku, Tokyo, 169-0073, Japan; Department of Pathology, Tokyo Yamate Medical Center, 3-22-1 Hyakunin-cho, Shinjuku-ku, Tokyo, 169-0073, Japan; Department of Pathology, Dokkyo Medical University, 880 Kitakobayashi Shimotsugagun Mibumachi, Tochigi, 321-0293, Japan

**Keywords:** thymoma, unencapsulated, microthymoma

## Abstract

Thymomas, the most common mediastinal tumors, form capsules. Only a few reports have presented small thymomas without capsule formation, so-called microthymomas. Here, we report a case of an unencapsulated thymoma measuring 18 mm. A 42-year-old female presented with an anterior mediastinal tumor. Computed tomography revealed an 18-mm nodule in the anterior mediastinum. Magnetic resonance imaging revealed a solid tumor that was iso-intense on T1-weighted images and hypo-intense on T2-weighted images. Thoracoscopic partial thymectomy was performed. The histopathological diagnosis was a type B1 thymoma. The tumor was localized within the thymic tissue lacked a fibrous capsule and partially invaded the surrounding fat tissue. To our knowledge, this is the first report of an unencapsulated thymoma, except for microthymomas.

## Introduction

Thymomas are the most prevalent mediastinal tumors that form capsules. Only a few studies have reported incidentally detected small, unencapsulated thymomas, known as microthymoma [1]. Here, we describe a case of an 18-mm unencapsulated thymoma, which is the first report of an unencapsulated thymoma measuring > 10 mm.

## Case report

**Figure 1:**
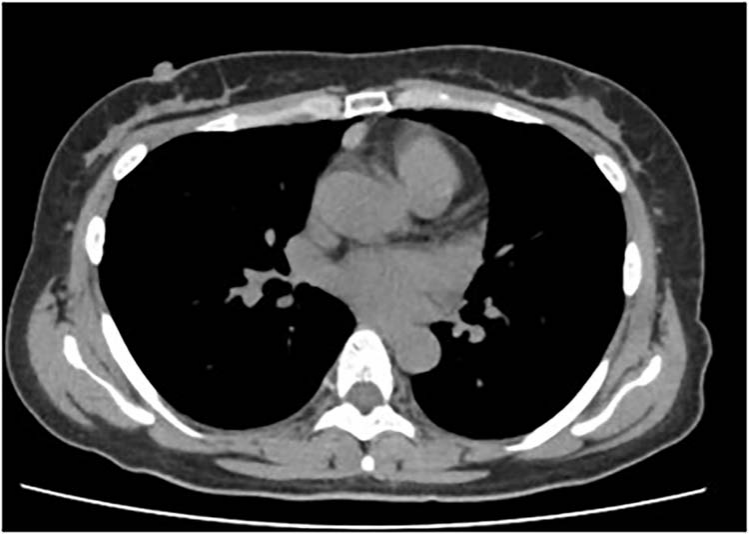
Chest CT showing a well-circumscribed tumor shadow of 15 mm in the anterior mediastinum.

**Figure 2:**
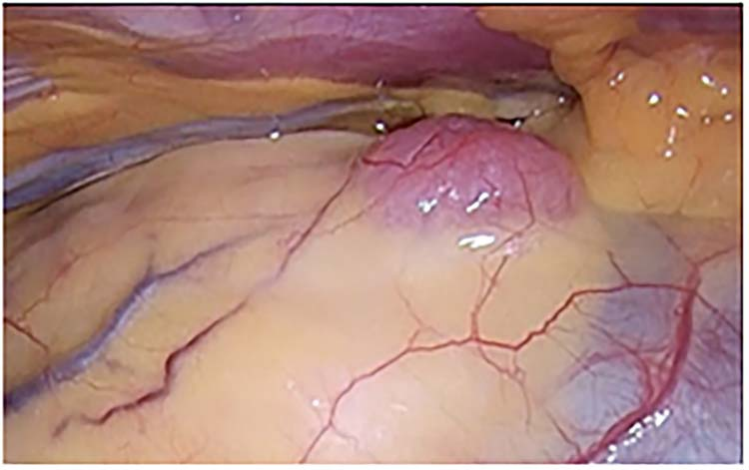
A pale red tumor covered with pleura at the edge of the right lower pole of the thymus.

**Figure 3:**
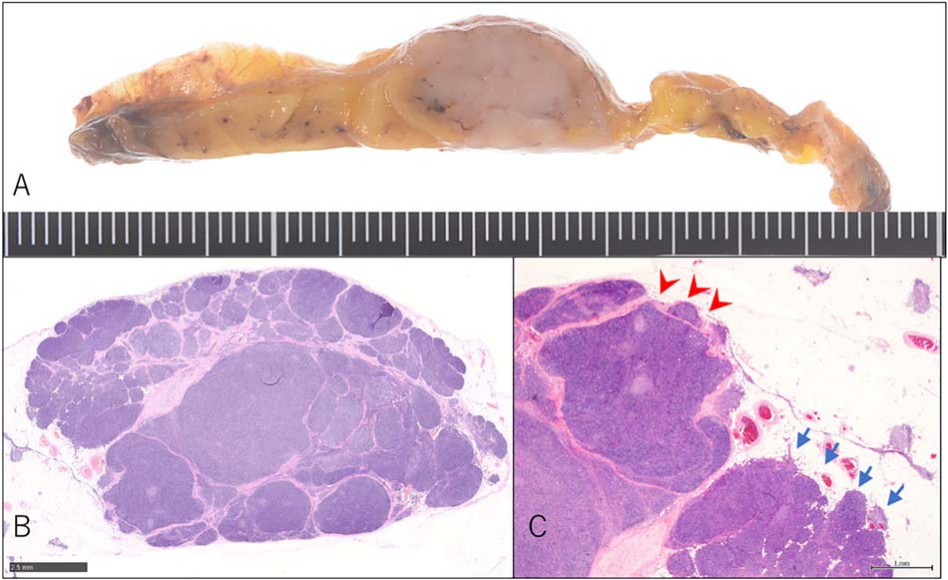
Pathological findings. (A) Macroscopic findings of the resected specimen: lobular whitish tumor below the pleura. (B) Microscopic findings of the resected specimen on hematoxylin–eosin staining. Type B1 thymoma detected just above the pleura without entire fibrous capsule formation (magnification: bar = 2.5 mm). (C) Short fibrous band at the edge of the tumor (arrow-head), whereas invasion of the surrounding thymus lacked fibrous capsule (arrow) (magnification: bar = 1 mm).

An asymptomatic 42-year-old female, with a history of ulcerative colitis and posterior longitudinal ligament ossification, had a mediastinal tumor detected incidentally on magnetic resonance imaging (MRI) and was admitted to our hospital for surgical intervention. No findings such as ptosis or muscle weakness were observed. Chest non-contrast computed tomography (CT) scans showed a well-circumscribed tumor shadow with a diameter of 18 mm in the anterior mediastinum (Fig. 1). Thoracic MRI revealed a homogeneous tumor with iso-intensity on T1-weighted and low-intensity on T2-weighted imaging. The tumor markers alpha-Fetoprotein, human chorionic gonadotropin, and carcinoembryonic antigen were within normal ranges. Serum anti-acetylcholine receptor antibody levels were normal. The patient underwent partial thoracoscopic thymectomy. The tumor, located at the edge of the right lower pole of the thymus, was smooth, slightly solid, and covered with mediastinal pleura. The pale red color of the tumor was visible from the surface through the pleura, indicating that it was located in superficial layer close to the pleura (Fig. 2). The tumor was resected along with the surrounding thymus with a sufficient resection margin. Gross examination revealed an 18 × 15 mm lobular whitish solid tumor below the pleura (Fig. 3A). Microscopic examination revealed that the tumor was composed of epithelial-like cells with significant lymphocytic infiltration and was divided into several lobules by fibrous septa. However, mitosis was rarely observed (Fig. 3B). The formation of the fibrous capsule was incomplete, and intermittent fibrous tissue was observed around the tumor (Fig. 3C). The surgical margins were negative. The tumor was pathologically diagnosed as a type B1 thymoma [World Health Organization (World Health Organization: WHO) classification], T1aN0M0, Stage I (TNM classification 8th edn), and Stage II, according to Masaoka–Koga staging. The patient was discharged on postoperative Day 7 without any complications. As curative resection was achieved, adjuvant therapy was not planned. Our patient has been followed up for 18 months postoperatively, with no recurrence.

## Discussion

Thymomas are the most common type of thymic neoplasm, with an incidence ranging from 0.13 to 0.26 cases per 100 000 population per year, and occur slightly more frequently in females [2]. Thymomas are well defined, round or lobulated, and homogenous. On gross examination, these tumors have a surrounding capsule, which may be associated with invasion of the surrounding organs (pleura, pericardium, or lung). Therefore, capsule formation is considered an essential factor in tumor staging in either the TNM system [3] or the Masaoka–Koga system [4]. In the TNM system, an encapsulated tumor and microscopic transcapsular invasion are classified as T1a, whereas in the Masaoka–Koga classification, an encapsulated tumor is classified as Stage I, and a microscopic transcapsular invasion is classified as Stage II. In the present case, no fibrous tissue was observed around the thymoma, and only the pleura covering the thymus was observed above the tumor. The tumor was localized and had partially invaded fatty tissue but had not invaded the surrounding organs; thus, the diagnosis was T1b using TNM staging and Stage II using Masaoka–Koga staging.

Reports on unencapsulated thymomas are limited to microscopic thymomas and microthymomas. Microscopic thymoma was first described by Rosai and Levine in 1976 as a small thymic epithelial cell nest measuring < 1 mm, with or without a capsule. However, this term was removed in the 5th edn of the WHO classification as it is unlikely to be neoplastic [5]. In contrast, the term microthymoma was defined in 2007 by Cheuk *et al*. as a small tumor with a complete thymoma morphology. They reported a case of a 7-mm thymoma lacking a fibrous capsule [1]. Three cases of unencapsulated microthymomas have been reported, including one reported by Cheuk *et al*. Shimosato *et al*. [6] illustrated a 5-mm microthymoma, and Wada *et al*. [7] reported an 8-mm microthymoma. In Cheuk *et al*. and Wada *et al*.’s reports, these tumors were incidentally identified in resected specimens of patients with coexisting diseases (pure red cell aplasia and myasthenia gravis, respectively). Thus, the present case was unique because the patient had no comorbidities, and the tumor diameter was larger than that in any other previously reported case.

Capsule formation in tumors is presumed to be a host defense mechanism and represents the growth characteristics of the tumor. The slower the tumor grows, the thicker and more complete is the capsule formation [6]. This phenomenon can be observed in several benign tumor growths that press against the surrounding tissue, resulting in capsule formation between the tumor and surrounding tissue [8]. Although the process of capsule formation in thymomas remains unknown, Cheuk *et al*. described the process of thymoma development by citing two different cases of microthymomas, one with a well-developed fibrous capsule and the other with no capsule. They determined that the fibrous capsule was not present at the inception of the thymoma but developed at an early stage [5]. Therefore, the lack of a capsule around the tumor in our case suggests that it was in the early stages of thymoma development. Because the tumor diameter was >10 mm, it may have grown in a relatively short time, but this could not be confirmed owing to the lack of previous CT images. However, histological findings revealed that the tumor was lobulated by a fibrous septal wall and mixed with tumor components at its borders, indicating progressive capsule formation.

To our knowledge, this is the first report of a rare case of unencapsulated thymoma, except for microthymomas. Further case accumulation may provide insights into the developmental processes of thymomas.

## Data Availability

The data underlying this article will be shared on reasonable request to the corresponding author.
